# Tweedie distributions for fitting semicontinuous health care utilization cost data

**DOI:** 10.1186/s12874-017-0445-y

**Published:** 2017-12-19

**Authors:** Christoph F. Kurz

**Affiliations:** Institute of Health Economics and Health Care Management, Helmholtz Zentrum München, German Research Center for Environmental Health (GmbH), Neuherberg, Germany

**Keywords:** Health economics, Tweedie distribution, Health care utilization, Cost data

## Abstract

**Background:**

The statistical analysis of health care cost data is often problematic because these data are usually non-negative, right-skewed and have excess zeros for non-users. This prevents the use of linear models based on the Gaussian or Gamma distribution. A common way to counter this is the use of Two-part or Tobit models, which makes interpretation of the results more difficult. In this study, I explore a statistical distribution from the Tweedie family of distributions that can simultaneously model the probability of zero outcome, i.e. of being a non-user of health care utilization and continuous costs for users.

**Methods:**

I assess the usefulness of the Tweedie model in a Monte Carlo simulation study that addresses two common situations of low and high correlation of the users and the non-users of health care utilization. Furthermore, I compare the Tweedie model with several other models using a real data set from the RAND health insurance experiment.

**Results:**

I show that the Tweedie distribution fits cost data very well and provides better fit, especially when the number of non-users is low and the correlation between users and non-users is high.

**Conclusion:**

The Tweedie distribution provides an interesting solution to many statistical problems in health economic analyses.

## Background

In modelling cost data of health care utilization, the non-negative response variable is often zero because of non-users, while the positive realisations are usually usually right-skewed. Such variables are called *semicontinuous* [1] and pose a number of problems: because of the point mass at zero, common models involving the Gamma or log-normal distributions have difficulty with such a mixture of discrete and continuous values. A popular way to account for this in the generalized linear models (GLM) framework is the use of two-part models [2], which combine a binary model for the dichotomous event of having either zero or positive values with a continuous model for those having positive values. This complements a two-stage decision process, which can be inadequate because the two decisions are not usually made independently (Winkelmann [3] and Van Ophem [4] discuss this for the case of physicians visits). Another more simple model, using a single distribution, is the Tobit model [5]. This model is based on a zero-truncated normal distribution but cannot handle *excess zeros*, i.e. the presence of more zeros in the data than would be expected from the underlying distribution. In this linear regression setting, constant variance is assumed, which is also inadequate for cost data. Sometimes, count data model like the Poisson are also used for cost modelling [6].

Recent research has mainly focused on developing new models and comparing distributions for the continuous part of the two-part models. For example, the generalized Gamma distribution (GenG) is a flexible choice as it has one scale and two shape parameters. The standard Gamma, Weibull, exponential, and the log-normal are all special cases of this distribution. Manning finds that this distribution provides a more robust alternative estimator than the standard alternatives [7]. Jones et al. compared several recent developments in parametric and semiparametric regression models for health care costs [8]. Other comparative studies, in which models are compared on either real data, i.e. the true distribution is unknown, or using simulations, include Basu et al. [9], Hill and Miller [10] and Jones et al. [11]. They all focus on the analysis of positive costs with no emphasis on the zero aspect. The only comparative study considering zero costs is Buntin and Zaslavsky [6].

In this study, I consider a single distribution GLM for cost data that can simultaneously model the zeros and continuous positive outcomes. The number of excess zeros can be arbitrarily high while still providing good support for the positive costs. Variance can be specified as some power of the mean. This model, based on the family of Tweedie densities [12], has already been shown to perform well in the case of rainfall precipitation [13] and insurance premiums [14]. To my knowledge, the Tweedie densities have not been used in health economic cost data modelling before. In the following, I compare the Tweedie model with the two-part (Binomial/Gamma and Binomial/GenG), the Tobit, and the Poisson models regarding marginal effects (at the means), model fit and prediction error in both Monte Carlo simulation and real data. As analysts favour simple models that are easy to interpret, I restrict myself to these alternatives. For an overview of other, more specialized methods, I refer to Mihaylova et al. [15] and the literature already mentioned.

The rest of this paper is structured as follows: “Methods” section illustrates the properties of the Tweedie family of distributions and explains the proposed model. Furthermore, it outlines the simulation study and describes the data. The “Results” section compares the Tweedie with the two-part, Tobit, and Poisson models on these data before the last section concludes.

Code and data to reproduce all analyses are available on the author’s github page (https://git.io/v6adW. Accessed 16 Aug 2016).

## Methods

### Tweedie family densities

I outline the model used in this paper as a special case of exponential dispersion models (EDMs) [12]. This class of models is a broad family of distributions defined by the form

$$f(y|\theta, \phi) = a(y, \phi) \exp \left[ \frac{y\theta - \kappa(\theta)}{\phi} \right], $$

where both the normalizing functions *a*(·) and *κ*(·) are known. *θ* is the natural parameter and *ϕ*>0 is called the dispersion parameter. Mean *μ* and variance of a random variable *Y* from an EDM are given by *E*(*Y*)=*μ*=*κ* ^′^(*θ*) and Var(*Y*)=*κ* ^″^(*θ*)*ϕ* respectively. The Tweedie family of distributions corresponds to special cases of EDMs where the power mean-variance relationship is characterized by Var(*μ*)=*ϕ* *μ* ^*p*^ for *p*∉(0,1). The Tweedie family includes a number of familiar distributions, e.g. Normal (*p*=0), Poisson (*p*=1), Gamma (*p*=2) and inverse Gaussian (*p*=3).

For cost data modelling, the choice *p*∈(1,2) is the most interesting one and the main focus here because of its support for semicontinuous outcomes. Tweedie distributions in this range of *p* belong to the so-called compound Poisson-Gamma distributions [12]. Let *M*∼Pois(*λ*) be a Poisson random variable and let *X*_*i*_∼*i* *i* *d*Gamma(*α*,*β*) be Gamma distributed with *M*⊥*X*_*i*_, then a random variable *Z*, defined by

$$Z=\left\{\begin{array}{ll} 0, & \text{if }M=0,\\ X_{1}+X_{2}+{\ldots}+X_{M}, & \text{if }M=1,2,{\ldots} \end{array}\right., $$

follows a compound Poisson-Gamma distribution, i.e. is a Poisson sum of Gamma random variables. If *M*=0, then *Z*=0, thus allowing for a probability mass at zero for non-users, where Pr(*Z*=0)= exp(−*λ*). If *M*>0, then *Z* is the sum of *M* iid Gamma random variables, so conditional on *M*, *Z*|*M*∼Gamma(*M* *α*,*β*), resulting in a continuous distribution for the positive outcome. With *M*=*m*, the distribution for *z*>0 is therefore given as:

$$f(z|\lambda, \alpha, \beta) = \frac{\lambda^{m} \exp(-\lambda)}{m!} \frac{z^{m\alpha-1}\exp(-z/\beta)}{\beta^{m\alpha}\Gamma (m\alpha)}. $$

These parameters *λ*,*α* and *β* are related to the Tweedie distribution parameters *μ*,*ϕ* and *p* by:

$$\lambda = \frac{\mu^{2-p}}{\phi(2-p)}, \quad \alpha = \frac{2-p}{p -1}, \quad \beta = \phi(p-1)\mu^{p-1}. $$

Recovering the underlying marginal distribution of *Z* results in a non-closed form expression for the normalizing function *a*(·), based on Wright’s generalized Bessel function *W*(·,·,·) [13, 16]:

$$a(z,\phi)=\frac{1}{y}W(z,\phi,p). $$

Dunn and Smyth [17] show that this function is strictly convex and can be approximated by Stirling’s formula for the Gamma function and a Fourier inversion method for the infinite series. In practice, first, the parameters *ϕ* and *p* are estimated by numerically maximizing the profile likelihood, i.e. profiling out the mean parameter *μ* as it is determined for a given value of *ϕ*. Second, the mean parameter is estimated using a GLM with the previously estimated *ϕ*. The Tweedie distribution cannot be expressed in closed form. To compute the profile likelihood, numerical optimization methods must be used [16].

Because Tweedie distributions also belong to the exponential family of distributions, they can be used in the GLM framework [18]. Besides the ability to model exact zeros and continuous outcomes, the idea that positive total costs are sums of smaller costs provides an intuitive appeal: *Z* is the total amount of expenses in a given period, *M* the number of utilization events, and *X*_*i*_ the expenses of the *i*-th event. In the following, I show that the Tweedie distributions fit health care utilization cost data very well.

### Monte Carlo simulation

I used a Monte Carlo simulation to address two common situations when modelling semicontinuous cost data:

1. The non-users can be substantially different from the users, i.e. they imply different characteristics and have *low correlation* with the users.
2. The non-users belong to the same “distribution” as the users, i.e. they share the same personal attributes, and therefore show *high correlation* with the users.

In both situations, a broad range of circumstances that are common in health care cost data were examined. They are: (1) skewness and non-normality of the costs; (2) range of the positive costs; and (3) outliers, i.e. proportion of individual high cost cases. The Gamma distribution provides a way to deal with these matters flexibly by specifying the shape and rate parameters. In the GLM, the outcome *Y* of the dependent variables is generated from a distribution in the exponential family. The mean *μ* of the distribution depends on the independent variables, *X*, through

$$ E(Y) = \mu = g^{-1}(X\beta),$$

where *E*(*Y*) is the expected value of *Y*, *X* *β* is the linear predictor and *g* is the link function. The probability density function of the Gamma distribution with shape parameter *α*>0 and rate parameter *θ*>0 is defined by

$$f(x; \alpha, \theta) = \frac{\theta^{\alpha}}{\Gamma(\alpha)} x^{\alpha-1} \exp(-\theta{x}), \quad x> 0.$$

The expectation of the Gamma distribution is $E(X)=\frac {\alpha }{\theta }$ and the variance is $Var(X)=\frac {\alpha }{\theta ^{2}}$. Generating the outcome *Y* given a linear predictor *X* *β* and a rate parameter *θ*, using a log-link function *g*(*z*)= log(*z*), is therefore possible because

$$Y \sim \text{Gamma}\left(\frac{\exp(X\beta)^{2}}{\theta}, \frac{\exp(X\beta)}{\theta}\right). $$

To generate the data for the Monte Carlo simulation, I built a data matrix *X* with three columns for covariates. I drew values in these columns at random from uniform distributions *U*(2,8), *U*(−10,1), and *U*(−2,0) respectively. Corresponding parameters *β*_1_,*β*_2_, and *β*_3_ were drawn from *U*(−2,1). Together with a rate parameter of *θ*=30, these choices allow for a wide variation of possible shapes of the Gamma distribution, and consequently of the costs *Y*.

In addition, I evaluated the following different proportions of non-users/zeros for both situations: 0.05, 0.1, 0.15, 0.2, 0.3, 0.5 and 0.7. This choice covers a broad range of settings that occur in real world scenarios. In the *low correlation* setting, I drew values for non-users from uniform distributions *U*(3,6), *U*(−2,3), and *U*(−1,1) to account for slightly different personal characteristics. In the *high correlation* setting, I set users corresponding to the lowest percentiles to zero. This implies that users and non-users share very similar characteristics but only differ in costs. For both low and high correlation and the varying amount of zeros, I generated 100 different data sets, each with *N*=5000 observations, which reflect a great diversity of possible scenarios. The choice of 5000 observations conforms to many real-world situations. Fewer observations often lead to numerical instabilities, especially in the presence of many zeros, while more observations usually do not improve the estimations.

As comparison metrics, I chose the Akaike information criterion (AIC) for model fit and the root mean square error (RSME) for predictive accuracy. RMSE is defined by

$$\operatorname{RMSE}=\sqrt{\frac{1}{N}\sum_{i=1}^{N} \left(\hat y_{i} - y_{i}\right)^{2}}, $$

where $\hat y$ denotes the estimate and *y* the true value. Because of the squared term, larger errors have a disproportionately large effect on RMSE. This effect is desirable when predicting cost data with outliers. For an unbiased estimator, the RMSE is the standard deviation. The reported value is the average of a 5-fold cross-validation. Cross-validation involves partitioning the data into five complementary subsets, performing the analysis on four subsets, and validating the analysis on the one remaining subset. This is repeated four times so that each subset is used once for validation, and the validation results are combined (e.g. averaged) over the rounds to estimate a final RMSE value. As both AIC and RMSE have no intrinsic interpretation for comparison across different data sets, I defined a summed rank value. In this ranking, the best model (lowest AIC or lowest RMSE) is assigned a value of 1, whereas the worst of the five compared models gets value 5. Ranks 2, 3, and 4 are assigned accordingly, resulting in a theoretical value of 100 for a model that wins across all 100 data sets.

### Data

In a second evaluation, I used data from the RAND Health Insurance Experiment (RAND HIE). This US study measured health care costs, among other outcomes, of people randomly assigned to different kinds of plans. Because of the random assignment, the reliability of health insurance coverage and the availability of important variables for this application, these data provide an accurate base for cost modelling in this case.

As outcome variable I took the total costs, consisting of outpatient, drug, supply, psychotherapy and inpatient expenses. I selected covariates commonly considered to determine health care utilization. Among the socio-economic characteristics were age, gender, race, the logarithm of family income (LINC), the number of physical limitations (PHYSLM), the number of chronic diseases (DISEA), the logarithm of family size (LFAM), the education of the household head in years (EDUCDEC) and a dummy variable indicating self-rated health as good (HLTHG). Insurance-specific variables included the log coinsurance rate plus 1 (LOGC), a dummy for the individual deductible plan (IDP), the log of the participation incentive payment (LPI), and a maximum expenditure function (FMDE). A more detailed description of the data set and the variables is available in Deb et al. [19].

I only chose the first year of observation for each individual 18 years and older (*N*=3301). There are 18.1% zero observations for the costs with a mean of 206.75 (standard deviation 597.98) and a maximum of 17730. The positive costs alone have a mean of 252.49 and a standard deviation of 652.05, indicating the skewness.

For model comparison on these data, I again computed AIC and RMSE (in 5-fold cross-validation). In addition, I looked at the marginal effects. The marginal effect of an independent variable is the derivative of a given function of the covariates and coefficients of the preceding estimation. The derivative is evaluated at the means of the covariates.

## Results

### Simulation study

**Fig. 1:**
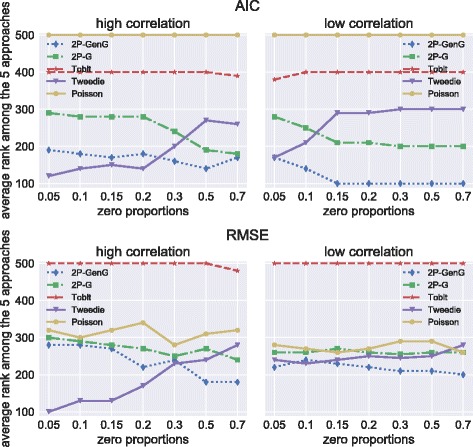
Rank values for AIC and RMSE for all models assessed in 100 simulated data sets each in situations with different percentages of zero costs. The best model (lowest AIC or lowest RMSE) is assigned a value of 1, the worst gets 5. Plots show the rank sums of 100 data sets; lower values are bette

In this section, I apply the two-part, the Tweedie, the Tobit, and the Poisson models to the simulated data and the RAND HIE data. The two-part model involves two estimations: first, it decides whether someone has zero or non-zero costs using a logistic regression. Second, conditional on having non-zero costs, it applies a continuous distribution to the positive outcome. I used both Gamma with log-link and generalized Gamma with log-link for this part. I also used the log-link for the Tweedie and the Poisson model. The Tobit model features an underlying normal distribution truncated at zero. For a more detailed description and justification of the two-part, Tobit, and Poisson models, see [1] and [15]. The aim of this application is to show how model choice affects model fit and prediction in the case of semicontinuous health care cost data. In the RAND HIE case, I also look at the marginal effects, but I reveal no causal effects in this study. Figure 1 presents the Monte Carlo simulation results of the rank comparison of both RMSE and AIC across the different settings with low and high correlation and varying numbers of zeros. If the number of zeros was below 20%, the Tweedie model outperformed the Tobit, the Poisson, and both two-part models in situations with high correlation between users and non-users. When the zero percentage was above 0.2, two-part models started to surpass the Tweedie model in both AIC and RMSE. The difference in performance regarding RMSE was less apparent between two-part and Tweedie models in the low correlation setting. However, two-part models had better model fit in these situations. The GenG was always better than the Gamma, whereas the Tobit performance was generally very bad. The Poisson model had very bad model fit but good predictive performance, especially in the low correlation setting.

### RAND HIE data

**Fig. 2:**
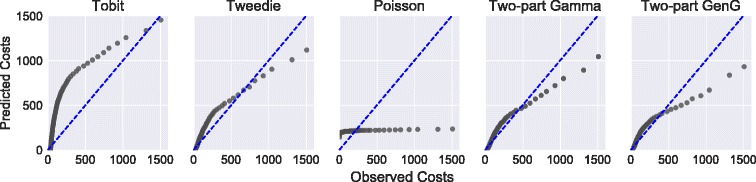
Q-Q plots for true and estimated quantiles of total health care utilization in the RAND HIE data for all models. Because of heavy outliers, I do not show the last percentile. Quantile values closer to the dashed line represent a better match of empirical and estimated distributions

Table 1 presents the marginal effects estimation results for the models discussed using the RAND HIE data. Although the estimates of the Tobit model are quite different in the value range and sign, the Tweedie and both Gamma and GenG parts of the two-part model are more similar: all estimates (except one for the GenG) shared the same sign and had comparable values, leading to similar conclusions. The Poisson model is more similar to the Tobit. Looking at the standard errors, both Tweedie and Gamma estimations lead to higher estimated standard errors than GenG. Furthermore, the AICs of the Tweedie and two-part Gamma models are almost identical, suggesting comparable model fit: the Tweedie AIC is 37777, whereas the two-part Gamma has a combined AIC of 37252. The two-part GenG shows superior model fit with a combined AIC of only 36209. The Poisson fits the data very badly with an AIC of 51495. The AIC of the Tobit model is significantly higher with a value of 43649. When plotting the true and estimated quantiles of the cost outcome for all distributions against each other, both two-part models exhibit better model fit for the lower quantiles, whereas the Tweedie model had slightly higher support for upper quantiles. See Fig. 2 for these Q-Q plots. This is probably because of the heavier tails of the Tweedie distribution. The Tobit model fits the central quantiles badly and the Poisson model is generally a bad fit.

**Table 1:** Comparison of marginal effects of Tobit, Tweedie, Poisson, and two-part (Binomial/Gamma and Binomial/GenG) models on the RAND HIE data

	Tobit		Tweedie		Poisson		Two-part					
							Binomial		Gamma		GenG	
	Est.	SE	Est.	SE	Est.	SE	Est.	SE	Est.	SE	Est.	SE
Intercept	-212.276	118.391	883.966	112.936	94.892	94.754	-0.091	0.050	1260.155	132.253	861.167	66.452
age	2.274	1.135	1.481	1.067	1.064	0.934	0.002	0.001	1.083	1.222	1.652	0.606
disea	6.319	1.695	3.045	1.566	3.534	1.408	0.005	0.001	2.127	1.806	5.664	0.895
physlm	214.712	34.484	143.054	31.272	191.965	28.998	0.040	0.019	168.177	36.817	118.515	18.349
logc	-23.994	17.530	-9.577	16.520	-8.445	13.323	-0.023	0.008	-3.963	19.191	-19.759	9.494
idp	-7.057	34.130	10.661	32.162	-3.311	27.925	-0.008	0.016	14.492	37.642	26.614	18.545
lpi	-1.806	5.612	-5.907	5.251	-1.625	4.612	0.001	0.003	-7.198	6.087	-4.135	3.005
fmde	1.728	10.453	3.828	9.846	-0.086	8.550	0.001	0.005	4.595	11.310	0.862	5.614
linc	18.113	11.951	13.757	11.492	6.876	9.452	0.012	0.005	8.076	13.893	13.392	6.926
lfam	-12.640	23.245	-2.754	21.955	-15.670	18.987	0.007	0.011	-1.910	25.514	-22.502	12.599
female	138.240	26.417	82.600	25.001	66.654	21.586	0.116	0.013	68.471	28.453	71.842	14.100
black	-166.908	39.181	-72.274	37.161	-60.124	31.299	-0.129	0.016	-28.317	44.221	-85.676	22.035
educdec	0.150	4.556	-2.301	4.290	-2.869	3.736	0.005	0.002	-4.205	4.897	1.894	2.441
hlthg	-17.324	25.583	-13.963	24.077	-23.013	20.990	0.013	0.012	-20.765	27.486	-18.554	13.627
*p*			1.719				2		2			
*AIC*	43649		37777		51495		2770		34482		33439	
*RMSE (5-CV)*	573.26		568.14		572.33			568.60		568.23		

*p* is the estimated mean-variance power parameter

Regarding RMSE, evaluated in a 5-fold cross-validation, two-part and Tweedie models again produce very similar results. Tweedie has the lowest RMSE with a value of 568.14, two-part Gamma has 568.60, and two-part GenG has 568.23. Tobit is slightly higher with a value of 573.26. The Poisson is slightly better than the Tobit with an RMSE of 572.33.

**Fig. 3:**
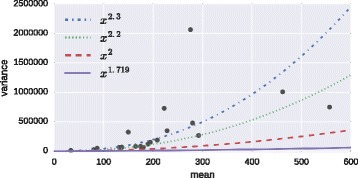
Mean-variance plots for all 5% quantiles for the Tweedie model. The solid line represents the estimated value for the mean-variance power parameter *p*=1.719. Other values are plotted for comparison

The estimated value for the mean-variance power parameter in the Tweedie model is *p*=1.719. Figure 3 shows the mean-variance plots for all 5% quantiles for the Tweedie model on the example data.

## Discussion

This paper explores a single distribution GLM based on the Tweedie family of distributions for semicontinuous cost data. This model is comparable in model fit to the two-part Binomial/Gamma and Binomial/GenG model but only includes a one-stage decision process, making it easier to interpret. The Tweedie model outperforms the Tobit model as the popular single distribution model for non-negative continuous data with a support for exact zeros. The Tweedie model further outperforms the Poisson model that is often used for cost data modelling despite being a count data model. Thus, the Tweedie model provides an interesting alternative for modelling of health care utilization cost data as it has natural support for cases in which no utilization has occurred and for those it has. The Tweedie model especially shines when the correlation between users and non-users of health care utilization is high and the proportion of these non-users is low. On the other hand, more sophisticated models such as the two-part Binomial/GenG show superior model fit and predictive accuracy when the proportion of zeros is high and the users and non-users suggest different characteristics. There exist situtations, especially when analysing inpatient utilization, where more that 70% zeros occurs. This is not covered in the simulation study. The simulations study only covers the RMSE metric for predictive accuracy. Other studies also measure the mean absolute error, or the mean error [6]. In the present case, this lead to almost identical results, presumably because of the ranking system. On the RAND HIE data, the Tweedie model shows slighly better predictive accuracy but worse model fit. The difference in RMSE was very small. Previous comparative studies including zero observations also found only small differences in predictive accuracy measures among different models [6]. To rule out unfortunate random splits in the cross-validation, I performed additional analyses with different random splits. No changes in the ranking occured and only insignificant changes in the RMSE values. The theoretical justification for the Tweedie model is given as, for the discussed case where the power parameter *p*∈(1,2), the Tweedie model can be explained as a Poisson sum of Gamma distributions. There, the number of utilization events is expressed by a Poisson distribution and the amount of each utilization by a Gamma distribution.

There exists a variety of models that were not included in this comparison. A prominent example is the extended estimating equations (EEE) model, which starts with estimates from a Gamma GLM and then iteratively improves the link and distribution functions based on the data [20]. This semiparametric model showed good performance in many comparison studies and has the advantage of omitting the need to specify a link function in advance. However, because of its iterative procedure, the EEE needs a large number of observations (usually more than 5000) to be efficient and, in the RAND HIE case and several simulated data sets, it did not converge. Also, it does not provide a closed form likelihood, and it is therefore unclear how the AIC can be used as a comparison metric. Jones et al. [8] use the Pearson correlation test for model comparison; because of various problems accompanying hypothesis tests in general [21] and the restriction to more simple parametric models, the AIC seems preferable. While the Pearson test can only summarize the specification of the conditional mean function on the scale of interest, the AIC measures goodness of fit of the whole distribution on the scale of estimation [8]. Jones et al. also find that the commonly used log-link is often not the ideal link function [8]. The Tweedie model also supports the inverse, identity and square root link function, but I could not observe any major deviations in the results when using these instead of the log. Even more so, link functions other than the log were more unstable in the maximum likelihood estimation and led to estimation errors, but this is probably an implementation issue in the numerical maximum likelihood optimization procedure.

Some other studies have used a quasi-Monte Carlo design for comparison studies [8, 22]. In this setting, several estimation data sets of various sizes are drawn with replacement from a large real data set and are then evaluated on a hold-out validation set. This was inappropriate in this analysis because I was implicitly interested in different relations between users and non-users and the varying amount of zeros. However, one disadvantage of the Monte Carlo simulation in this case is that only a predefined distribution, here the Gamma, can be used to generate data. This may bias the analysis towards models that use the Gamma, but the results show no evidence of that.

Recent research in health economic cost data modelling has mainly focused on the second continuous part of the two-part models. In this analysis, I show that the Binomial part may need more attention, as it is obvious that a logistic regression cannot adequately distinguish the non-users and users if they share similar characteristics (i.e. they are highly correlated) and the classes are very unbalanced (i.e. the number of non-users is low).

Although the theory of the Tweedie families has been known for more than 20 years, only recently have software packages that allow easy handling of these distributions become available [16, 23]. Further research should explore the usefulness of Tweedie distributions with *p*>2 as they provide similar shape to the Gamma but support heavier tails. Tweedie models in this range may be an attractive alternative for the continuous part of a two-part model or for cases without exact zeros and support a more flexible mean-variance relationship. The estimated mean-variance power parameter *p*=1.719 may not appropriately reflect the true relationship. The fixed *p*=2 in Gamma models is still too low, but values of *p* in the range of 2.2 to 2.3 seem to be more realistic when visually comparing the curves in Fig. 3. This also has potential for further investigation.

Swallow et al. [24] show in an ecological setting that a Bayesian hierarchical model based on the Tweedie densities provides further flexibility and removes this need to make strong assumptions about mean-variance relationships a priori. Such a hierarchical extension may also be useful to account for correlated effects by repeated measurement of individuals, for example in clinical trial settings or claims data.

## Conclusion

Models based on Tweedie distributions are an interesting alternative for the analysis of semicontinuous health care cost data. They are especially useful when the correlation between users and non-users of health care utilization is high and the proportion of these non-users is low.

## Abbreviations

AIC: Akaike information criterion
EDM: Exponential dispersion model
GenG: Generalized Gamma distribution
GLM: Generalized linear model
RMSE: root mean square error
